# *Verticillium longisporum* infection induces organ-specific glucosinolate degradation in *Arabidopsis thaliana*

**DOI:** 10.3389/fpls.2015.00508

**Published:** 2015-07-10

**Authors:** Katja Witzel, Franziska S. Hanschen, Rebecca Klopsch, Silke Ruppel, Monika Schreiner, Rita Grosch

**Keywords:** glucosinolate breakdown products, natural variation, plant root, plant secondary metabolites, vascular pathogen

## Abstract

The species *Verticillium* represents a group of highly destructive fungal pathogens, responsible for vascular wilt in a number of crops. The host response to infection by *Verticillium longisporum* at the level of secondary plant metabolites has not been well explored. Natural variation in the glucosinolate (GLS) composition of four *Arabidopsis thaliana* accessions was characterized: the accessions Bur-0 and Hi-0 accumulated alkenyl GLS, while 3-hydroxypropyl GLS predominated in Kn-0 and Ler-0. With respect to GLS degradation products, Hi-0 and Kn-0 generated mainly isothiocyanates, whereas Bur-0 released epithionitriles and Ler-0 nitriles. An analysis of the effect on the composition of both GLS and its breakdown products in the leaf and root following the plants’ exposure to *V. longisporum* revealed a number of organ- and accession-specific alterations. In the less disease susceptible accessions Bur-0 and Ler-0, colonization depressed the accumulation of GLS in the rosette leaves but accentuated it in the roots. In contrast, in the root, the level of GLS breakdown products in three of the four accessions fell, suggestive of their conjugation or binding to a fungal target molecule(s). The plant-pathogen interaction influenced both the organ- and accession-specific formation of GLS degradation products.

## Introduction

A characteristic feature of Brassicaceous plants is the presence of glucosinolates (GLS), a group of sulfur-containing secondary metabolites which contribute to the plant’s defense against a range of biotic stresses (Agerbirk et al., 2009; Textor and Gershenzon, 2009). While GLS content is typically up-regulated by pathogen or pest attack, other forms of stress, notably UVB radiation (Mewis et al., 2012b) and drought (Mewis et al., 2012a) can also induce their accumulation. The 130 known GLS variants have been classified, according to the nature of the side chain present, into the aliphatic, aromatic and indole GLS (Agerbirk and Olsen, 2012). GLS are found in the vacuole; when the cell is disrupted, they interact with myrosinase to form either ITCs or nitriles. If the epithiospecifier protein (ESP) is present, the degradation process of alkenyl GLS generates an epithionitrile. The ESP protein has been also identified to favor the formation of nitriles from other (non-alkenyl) GLS (Wittstock and Burow, 2010; Kissen et al., 2012). In *Arabidopsis thaliana*, a number of other modifiers is known (Zhang et al., 2006; Burow et al., 2009; Kissen and Bones, 2009). Isothiocyanates (ITCs) act as an effective deterrent against many pathogens, including fungi, bacteria and even insects (Shofran et al., 1998; van Ommen Kloeke et al., 2012; Witzel et al., 2013). Nitriles and epithionitriles are generally less bioactive (Shofran et al., 1998; Matusheski and Jeffery, 2001; Wittstock et al., 2003).

A number of fungi belonging to the species *Verticillium* induce plants to wilt when they invade the vascular system. They can be responsible for significant losses in both crop yield and quality. The species complex has a broad host range, infecting lettuce, olive, cotton, eggplant and tomato, among others (Daayf et al., 1995; Tsror et al., 2001; Dervis et al., 2009; Atallah et al., 2011; Jimenez-Diaz et al., 2012). These hemibiotrophic species colonize the plant root surface in response to the presence of specific root exudates, and after penetrating the cortex and endodermis, spread systemically via the xylem in the form of conidia (Fradin and Thomma, 2006; Zhou et al., 2006). Heavy colonization of the xylem can obstruct the transpiration stream, forcing the plant to form new xylem tissue (Reusche et al., 2012). One of the known host responses to *Verticillium* infection is an adjustment in the level and identity of secondary metabolites (Floerl et al., 2012; Iven et al., 2012; Obermeier et al., 2013; König et al., 2014). Linking diversity in secondary metabolite profiles (especially GLS) to the host/pathogen interaction is a science still in its infancy (Moore et al., 2014). In particular, little effort has been made to factor in crosstalk between the above and below ground parts of the plant in the context of defense against soil-borne pathogens.

A previous screen characterized GLS profiles of 19 *A. thaliana* accessions and related the growth-suppressive effect of volatile GLS breakdown products on *Verticillium longisporum* growth to the abundance of 2-propenyl ITC (2Prop-ITC) in a biofumigation assay (Witzel et al., 2013). Here, we extend this study to investigate the *in planta* influence of fungal colonization on GLS and their bioactive breakdown products. The effect on these traits of colonization by *V. longisporum* has been investigated, by comparing the performance of four accessions chosen to contrast not just with respect to their GLS (alkenyl GLS: Bur-0, Hi-0; hydroxyalkenyl GLS: Kn-0, Ler-0) and GLS breakdown product profiles, but also in the extent of their susceptibility to *V. longisporum* infection. We hypothesize that metabolic fingerprinting of a group of important defense compounds in *A. thaliana* accessions with quantitative variation in fungal tolerance provides new clues to understand tissue-specific GLS partitioning in response to pathogens.

## Materials and Methods

### Chemicals

2Prop-ITC, (≥99%), benzonitrile (≥99.9%), 3-butenenitrile (2Prop-CN, ≥98%), 4-pentenenitrile (3But-CN, ≥97%), 3-phenylpropanenitrile (2PE-CN, ≥99%) and sucrose-sodium nitrate media were purchased from Sigma-Aldrich Chemie GmbH, Steinheim, Germany; IAN, (≥98%) from Acros Organics (Fischer Scientific GmbH, Schwerte, Germany); 3-butenyl ITC (3But-ITC, ≥95%) and 4-pentenyl ITC (4Pent-ITC, ≥95%) were purchased from TCI Deutschland GmbH, Eschborn, Germany; 3-hydroxypropionitrile was purchased from Thermo Fischer Scientific, Erembodegem, Belgium; 4-(methylthio)butyl ITC (4MTB-ITC, ≥98%) was purchased from Santa Cruz Biotechnology, Heidelberg, Germany; 5-(methylsulfinyl)pentyl ITC (5MSOP-ITC) was purchased from Enzo Life Sciences GmbH, Lörrach, Germany; 1-cyano-2,3-epithiopropane (CETP), (≥95%) was purchased from Taros Chemicals GmbH Co. KG, Dortmund, Germany; 4-hydroxybenzyl GLS (≥97%), methylene chloride (GC Ultra Grade), Tris, EDTA, NaCl, CTAB, chloroform/isoamylalcohol (24:1), β-mercaptoethanol and phenol/chloroform/isoamylalcohol (25:24:1) from Carl Roth GmbH, Karlsruhe, Germany; acetonitrile (Ultra Gradient HPLC grade) was purchased from J.T. Baker, Deventer, The Netherlands and NaSO_4_ (≥99%) and methanol (>99.9) were purchased from VWR International GmbH, Darmstadt, Germany. Potato dextrose agar was purchased from Merck, Darmstadt, Germany. All solvents were of LC or GC-MS grade.

### Plants, Growing Conditions, and Fungal Inoculation

Seed of the *A. thaliana* accessions Bur-0, Hi-0, Kn-0 and Ler-0 (kindly provided by L. Westphal, Leibniz Institute of Plant Biochemistry, Germany), selected on the basis of their contrasting GLS profiles (Witzel et al., 2013), were germinated in soil and transplanted into quartz sand after 2 weeks. The plants were fed with a liquid nutrient formulation (Gibeaut et al., 1997) and exposed to an 8 h photoperiod provided by 300 μmol m^-2^ s^-1^ light at 20°C (light period)/18°C (dark period).

*V. longisporum* isolate 43-3 (Zeise and von Tiedemann, 2002, kindly provided by A. von Tiedemann, Georg-August-University, Germany) was maintained on potato dextrose agar plates, and mycelial suspensions were prepared as described by Witzel et al. (2013). Briefly, a flask containing 100 mL SSN medium was inoculated with six 5 mm diameter agar disks cut from the margin of an actively growing *V. longisporum* culture and incubated for 1 week. A further 200 mL SSN medium was added, and the culture maintained for two additional weeks. After mechanical blending, the fragmented mycelial suspensions were centrifuged and the pellets rinsed twice by resuspension in sterile tap water. Conidia were counted in a Thoma chamber to allow the concentration to be adjusted to 10^6^ mL^-1^. A 10 mL aliquot (or 10 mL water for mock inoculations) of the conidial suspension was poured over the surface of a pot containing the 2 weeks-old *A. thaliana* seedlings, which were then grown on for a further 4 weeks. Rosette leaves and roots were harvested, snap-frozen and ground to a powder, which was stored at -80°C until required for the extraction of DNA, proteins and metabolites.

Plant growth measurements were taken of the fresh weight of root and rosette of 20 plants of all four *A. thaliana* accessions, grown under control conditions or inoculated with *V. longisporum*, in three independent experiments. The relative growth rate was determined from the ratio between growth of control and inoculated plants. Analysis for statistical significance was done using Student’s *t*-test implemented in SigmaPlot 12.3 software (SPSS Inc., Chicago, IL, USA).

### DNA Isolation and Quantitative Real-Time PCR (qPCR)

Genomic DNA was isolated using the CTAB extraction method (Doyle and Doyle, 1990; Tinker et al., 1993). Aliquots of 200 mg powdered root material were homogenized in 500 μL CTAB [100 mM Tris-HCl pH 8, 1.4 M NaCl, 20 mM EDTA, 2% (w/v) CTAB, 0.2% (v/v) β-mercaptoethanol], then extracted by the addition of 200 μL chloroform/isoamylalcohol (24:1), After centrifugation, the DNA pellet was dissolved in 200 μL nuclease-free water and treated with RNase A (15 μL of 50 μg mL^-1^ RNase). Following a phenol/chloroform/isoamylalcohol (25:24:1) extraction, the DNA was precipitated in acidic ethanol (Gebhardt et al., 1989) and dissolved in 10 mM Tris-HCl/1 mM EDTA (pH 8). DNA concentration was derived via a Nano Drop ND-1000 spectrophotometer (Peqlab, Erlangen, Germany). The abundance of fungal DNA present in the DNA sample was estimated by a qPCR assay based on the primer pair VDS1/2 (5′-CAC ATT CAG TTC AGG AGA CGG A/5′-CCT TCT ACT GGA GTA TTT CGG) which amplifies a 521 nt stretch of *V. longisporum* DNA (Li et al., 1999). Detection of *V. longisporum* was performed as described in Witzel et al. (2013).

### Analysis of GLS and their Breakdown Products

The GLS composition of the *A. thaliana* leaf and root samples was determined as desulfo-GLS, using a slightly modified form of the Wiesner et al. (2013b) method. The modifications were as follows: the extraction was based on 10 mg of lyophilized plant material, and the internal standard was a 0.05 μmol aliquot of 4-hydroxybenzyl GLS. The various desulfo-GLS were separated by a UHPLC-DAD device (UHPLC Agilent 1290 Infinity System, Agilent Technologies, Böblingen, Germany) equipped with a Poroshell 120 EC-C18 column of dimension 100 mm × 2.1 mm containing particles of size 2.7 μm (Agilent Technologies). The solvent gradient was formed by water (A) and 40% acetonitrile (B), starting at 0.5% B for 2 min, rising to 49.5% B over the next 10 min, then held for a further 2 min, increased to 99.5% B over the course of 1 min and finally held for a final 2 min. The flow rate was 0.4 mL min^-1^ and the injection volume 5 μL. Desulfo-GLS were identified by comparing retention times and UV absorption spectra with those of known standards. Quantification was done at 229 nm via the internal standard (IST) 4-hydroxybenzyl GLS using the response factor (RF) of the GLS relative to 4-hydroxybenzyl GLS.

The quantification of GLS breakdown products was based on a GC-MS analysis, as described (Witzel et al., 2013; Piekarska et al., 2014), using an Agilent 7890A Series GC System (Agilent Technologies) equipped with an Agilent 7683 Series Autosampler, an Agilent 7683B Series Injector and an Agilent 5975C inert XL MSD. Analytes were separated using a SGE BP5MS column 30 m × 0.25 mm × 0.25 μM (VWR International GmbH, Darmstadt, Germany). The chosen instrument settings differed only slightly from those given by Piekarska et al. (2014): the temperature was set to 35°C for the initial 3 min, then raised to 100°C at 9°C min^-1^, where it was held for 3 min; the rest of the protocol was identical to that given by Piekarska et al. (2014). Molecular species were identified by their mass spectrum and retention time in comparison with those of authenticated standards and with literature data (Kjaer, 1963; Spencer and Daxenbichler, 1980). Analyte content was calculated using benzonitrile as IST and the RFs of CETP (RF = 1.66), 2Prop-ITC (RF = 1.71), 2Prop-CN (RF = 3.71), 3But-ITC (RF = 1.28), 3But-CN (RF = 2.61), 3-hydroxypropionitrile (RF = 7.12), 4MTB-ITC (RF = 0.53), 5MSOP-ITC (RF = 0.98), 3-hydroxypropionitrile (RF = 7.12), 2PE-CN (RF = 0.54), and IAN (RF = 0.35) relative to benzonitrile. For the commercially unavailable compounds, a response factor equal to that of the chemically most similar compound was assumed. Thus, other epithionitriles than CEPT itself were quantified at hand of the RF of CEPT; that of 3-hydroxypentene nitrile (2OH3But-CN) was based on that of 3But-CN (1.28), those of the breakdown products of the methylsulfinyl-alkyl GLS on that of 5MSOP-ITC (0.98), those derived from methylthioakyl GLS on that of 4MTB-ITC (0.53), the degradation products of 3-hydroxypropyl GLS on that of 3-hydroxypropionitrile (7.12), and that of 4-methoxy-3-indoleacetonitrile (4MO-IAN) on that of IAN (0.35). The limit of detection ranged between 0.2 μM (2PE-CN) and 10 μM (3-hydroxypropionitrile).

Because of the extent of the inter-experiment variation for metabolite concentrations, quantitative changes in GLS and their breakdown products induced by fungal colonization were analyzed separately for each of the three experiments. Relative fold changes between the inoculated and the non-inoculated plants were determined for each compound and the Student’s *t*-test was applied to identify statistically significant differences in mean compound content, applying a threshold of *p* < 0.05 (**Figures 3** and **4**). Absolute amounts of GLS and their respective breakdown products are presented in Supplementary Tables S1 and S2 as a mean of three independent experiments and the standard error.

Hierarchical clustering of profiles of GLS and GLS breakdown products was performed using MultiexperimentViewer MeV v4.7.4, based on Pearson correlation and average linkage clustering, on log2 transformed ratios between control and inoculated plants (Saeed et al., 2003).

To estimate the relation of total GLS breakdown products to the total amount of GLS, all values were converted to fresh weight basis. The recovery rate was determined as ratio between amount of total GLS breakdown products and amount of total GLS multiplied with 100% in three independent experiments. Analysis for statistical significance was done using Student’s *t*-test implemented in SigmaPlot 12.3 software (SPSS Inc., Chicago, IL, USA).

### Western Blotting

Proteins were extracted from the leaf and immunoblotted (based on 1 μg protein) as described by Amme et al. (2005). The blots were probed with an antibody recognizing the *A. thaliana* ESP, an enzyme responsible for diverting GLS hydrolysis from the generation of ITCs to that of epithionitriles or nitriles (Kissen et al., 2012, kindly provided by Ralph Kissen). Signal intensity was quantified using Phoretix 1D v11.4 gel analysis software (TotalLab, Newcastle upon Tyne, UK).

## Results

### Variation among the Four *A. thaliana* Accessions for Disease Susceptibility

**FIGURE 1:**
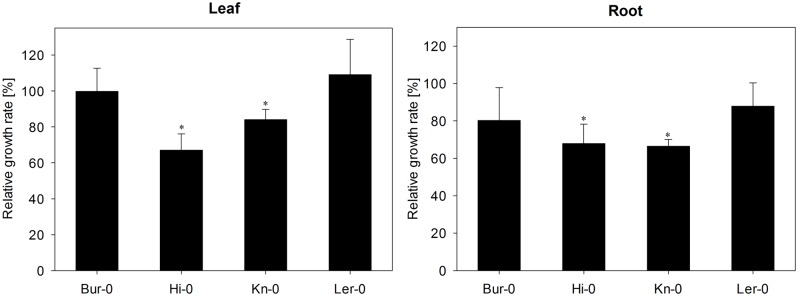
**The effect of *Verticillium longisporum* colonization on growth of *Arabidopsis thaliana* accessions Bur-0, Hi-0, Kn-0, and Ler-0.** Each data point represents the mean of three independent experiments. Relative growth rates were calculated by comparing inoculated with non-inoculated plants of each accession in turn on a fresh weight basis. The error bars denote the SE associated with the mean. Significant differences (*p* < 0.05) between inoculated and non-inoculated plants of the same accession are indicated by an asterisk.

**FIGURE 2:**
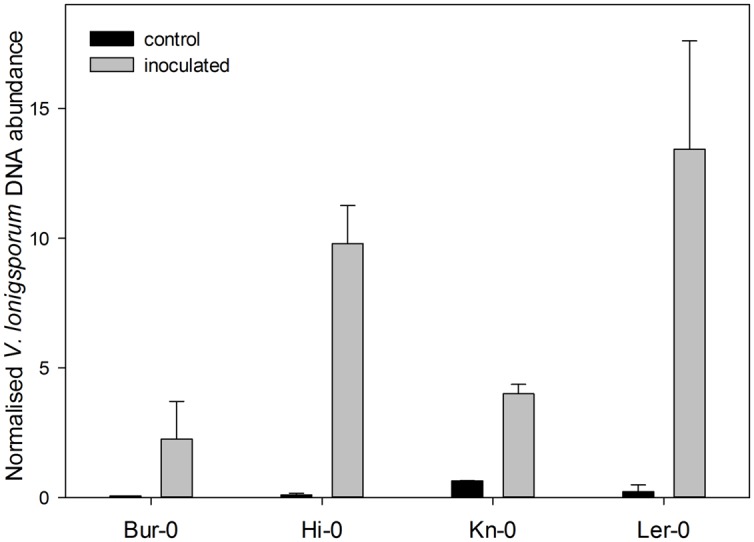
**The relative abundance of *V. longisporum* DNA in the roots of the four *A. thaliana* accessions, estimated via a qPCR assay carried out 28 days after inoculation.** Each data point represents the mean of three technical replicates and one representative experiment is shown. The error bars denote the SE associated with the mean.

The impact of the *V. longisporum* inoculation was stronger on the roots than on the leaf (**Figure 1**). All four accessions experienced a decline in root biomass, with Hi-0 and Kn-0 being the most severely affected. Leaf biomass was also reduced in both Hi-0 and Kn-0, while leaf biomass accumulation in Bur-0 and Ler-0 was relatively unaffected by the fungus. The only visible above-ground disease symptom observed at the time of harvest was the reduced growth of Hi-0 and Kn-0, but no wilting. Based on the biomass response to inoculation, Hi-0 and Kn-0 were classified as susceptible, and Bur-0 and Ler-0 as tolerant to *V. longisporum*. When the quantity of fungal DNA present in the root DNA preparation was tested by qPCR, the extent of the fungal colonization in the Ler-0 and Hi-0 roots was shown to be greater than in those of both Bur-0 and Kn-0 (**Figure 2**).

### The Effect of Fungal Colonization on GLS Profile

In all, 17 distinct GLS compounds were detected in the leaf material and 16 in the root of plants not infected with *V. longisporum*. While the overall GLS contents of the two organ types were rather different, their composition was quite similar, and in accordance with earlier data (Witzel et al., 2013). A full tabulation of the various GLS compounds identified is presented as Supplementary Table S1; this includes those compounds which were inconsistently present and therefore not analyzed further. Overall, there were 15 compounds reliably present in Bur-0, nine in Hi-0, eleven in Kn-0 and ten in Ler-0. The predominant GLS in the Hi-0 and Bur-0 leaf were the alkenyls 2Prop-GLS and 3-butenyl GLS (3But-GLS) in Hi-0 and Bur-0, respectively, while the commonest GLS in the leaf of Kn-0 and Ler-0 was 3-hydroxypropyl GLS (3OHP-GLS). The roots of Bur-0 contained substantial quantities of both 8-(methylsulfinyl)octyl GLS (8MSOO-GLS) and 1-methoxyindole-3-ylmethyl GLS (1MOI3M-GLS), while Hi-0 roots in addition featured 2Prop-GLS. In contrast, the GLS content of the Kn-0 and Ler-0 roots was dominated by 3OHP-GLS.

**FIGURE 3:**
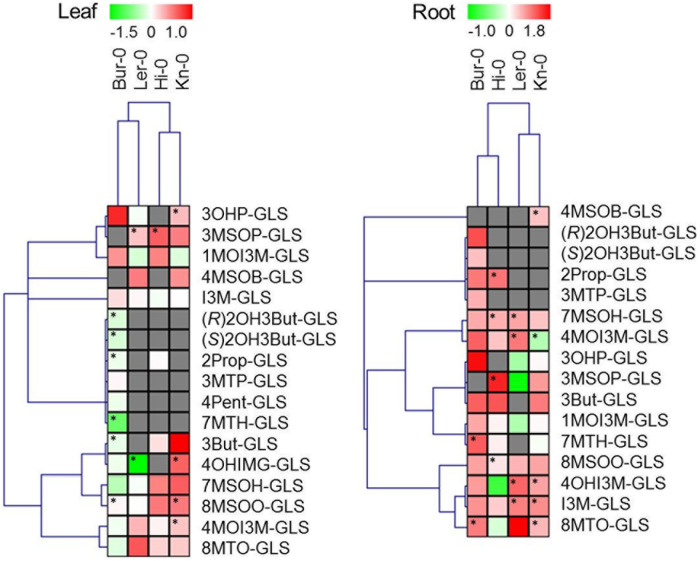
**Changes in the GLS profile induced by colonization with *V. longisporum*.** Each column represents a single accession and each row the fold change (inoculated/non-inoculated), calculated from three independent experiments and log2 transformed. Cells colored gray indicate the absence of the specific compound. Significant differences (*p* < 0.05) between inoculated and non-inoculated plants are indicated by an asterisk. Abbreviations are explained in the list of abbreviations.

In *V. longisporum* infected plants, the global GLS level was significantly higher than in the non-inoculated plants, especially in the root (**Figure 3**, Supplementary Table S1). For Bur-0, the root GLS content rose by 57%, while that of the leaf fell by 7%. Similarly, the root of Hi-0 accumulated 55% more GLS than the non-infected root, while its leaf GLS content rose by 7%. The effect of *V. longisporum* infection in Kn-0 was an 11% rise in root GLS content and a 20% rise in leaf GLS content. Ler-0 was the accession least affected by the presence of the fungus: its root GLS content was enhanced by 3% while its leaf GLS content dropped by 6%. With respect to GLS profile, in Bur-0 roots, all of the GLS species were more abundant than in the non-infected roots, while in its leaf, the representation of ten GLS species was reduced, suggestive of GLS translocation toward the site of infection. The behavior of Ler-0 was similar, featuring an increase in the presence of the indoles indole-3-ylmethyl GLS (I3M-GLS) and 4-hydroxyindole-3-ylmethyl GLS (4OHI3M-GLS) and the sulfinylalkyls 8MSOO-GLS and 7-(methylsulfinyl)heptyl GLS (7MSOH-GLS) in the infected root and a decrease in the leaf. In both Hi-0 and Kn-0, the abundance of most of the GLS compounds rose in both the root and the leaf. The attempt to compare the GLS profiles of Bur-0 and Ler-0 with those of Hi-0 and Kn-0 to identify which GLS species acted as a signature for *V. longisporum* tolerance was unsuccessful.

### The Effect of Fungal Colonization on the GLS Breakdown Product Profile

The four *A. thaliana* accessions differed from one another not only with respect to their GLS profile, but also with respect to the spectrum of GLS breakdown products generated (Supplementary Table S2). Unsurprisingly, given that Bur-0 contained the greatest diversity of GLS species, its degradation product spectrum was also the most diverse, comprising 22 products; the number for Hi-0 was 14, for Kn-0 eleven and for Ler-0 ten. The dominant species in the Bur-0 leaf were the epithionitriles 1-cyano-3,4-epithiobutane (CETB), derived from 3But-GLS, and 1-cyano-2,3-epithiopropane (CETP), derived from 2Prop GLS due to high ESP protein abundance (Supplementary Figure S1). In the Ler-0 leaf, the most abundant products were 4-hydroxybutylnitrile (3OHP-CN) and the corresponding 3-hydroxypropyl ITC (3OHP-ITC). The Hi-0 leaf contained mainly 2Prop-ITC and lacked CEPT, while 90% of degradation products from 3OHP-GLS was the corresponding ITC in leaves of Kn-0. No ESP was detected in the root of both Bur-0 and Ler-0 (data not shown), and as result, the root GLS degradation profiles were rich in ITCs.

**FIGURE 4:**
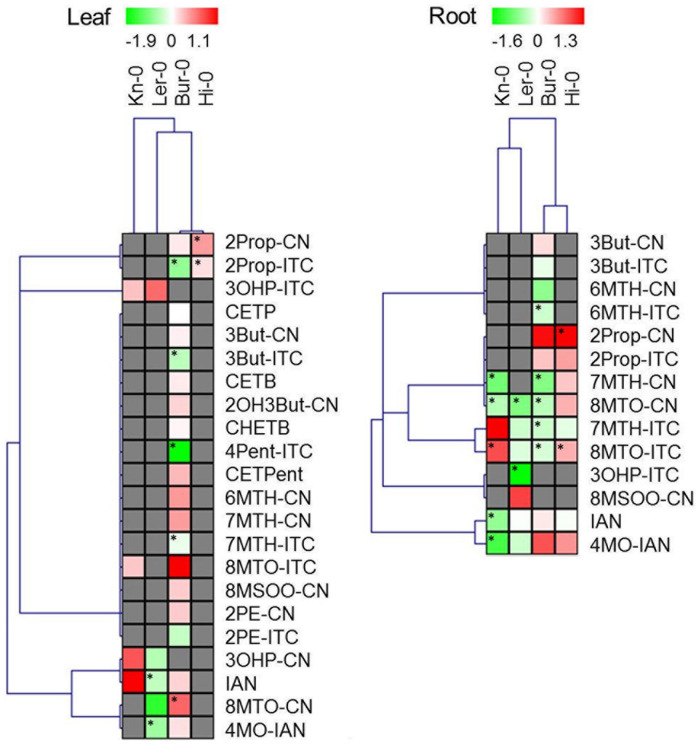
**Changes in the GLS breakdown product profile induced by colonization with *V. longisporum*.** Each column represents a single accession and each row the fold change (inoculated/non-inoculated) calculated from three independent experiments and log2 transformed. Cells colored gray indicate the absence of the specific compound. Significant differences (*p* < 0.05) between inoculated and non-inoculated plants are indicated by an asterisk. Abbreviations are explained in the list of abbreviations.

**FIGURE 5:**
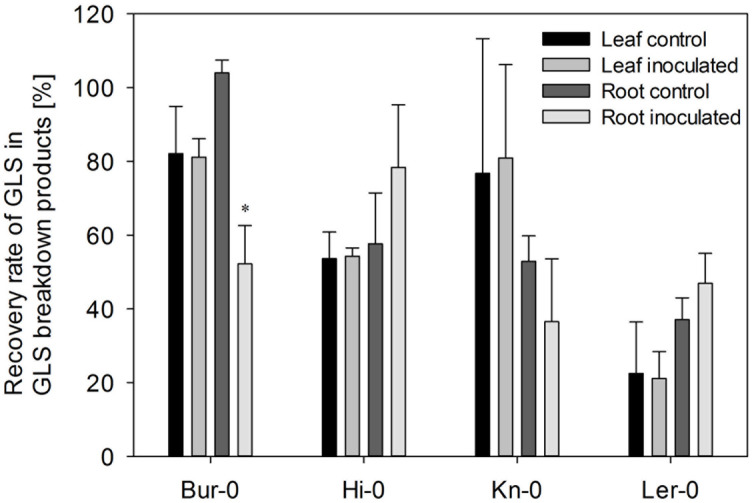
**The recovery rate of GLS in the form of breakdown products.** Each data point represents the mean of three independent experiments. The recovery rate was calculated from the ratio between the total GLS content and total GLS breakdown product content for each experiment, accession and treatment separately. The error bars denote the SE associated with the mean. Significant differences (*p* < 0.05) between inoculated and non-inoculated plants of the same accession are indicated by an asterisk.

Infection with *V. longisporum* altered the spectrum of GLS degradation products in an accession-specific manner (**Figure 4**). The Ler-0 leaf accumulated fewer nitriles, in accordance with its reduction in ESP abundance (Supplementary Figure S1). Although the abundance of ESP was reduced in the Bur-0 leaf, the formation of ITCs was less favored, while that of nitriles was enhanced. In both Kn-0 and Hi-0, the representation of both nitriles and ITCs increased. The quantity of GLS breakdown products in the leaf was enhanced by the fungal colonization in Bur-0, Hi-0 and Kn-0, but decreased in Ler-0. Three of the accessions (the exception was Hi-0) responded to infection by accumulating fewer detectable degradation products in their root. In the case of Hi-0, both ITC and nitrile levels were raised. Bur-0 and Ler-0 generated a reduced quantity of nitriles and of certain ITCs, while the Kn-0 profile involved an enhancement in ITC and a reduction in nitrile content. In Bur-0 and Ler-0, both the global GLS degradation product levels in the leaf and root were reduced upon inoculation: the reduction in the leaf was 7% in Bur-0 and 37% in Ler-0, while in the root the respective decreases were 17 and 30%. However, in Kn-0, the leaf global GLS degradation product content was increased by 16%, while that of the root fell by 21%. Finally in Hi-0, both the leaf and root content rose by, respectively, 11 and 53%, as a result of *V. longisporum* spread. Relative to that of GLS, the concentration of breakdown products derived from GLS lay between 21% in the leaf of Ler-0 plants inoculated with *V. longisporum* and 104% (non-inoculated Bur-0 roots; **Figure 5**). In response to *V. longisporum* inoculation, a significant change in the relative concentration was only noted in the roots of Bur-0, where a decrease of 50% was estimated.

## Discussion

The interaction between *Verticillium* and *A. thaliana* has been characterized at the level of host/pathogen signaling (Johansson et al., 2006; Pantelides et al., 2010; Tischner et al., 2010; Roos et al., 2014) and the genetic basis of host tolerance has been investigated (Veronese et al., 2003; Häffner et al., 2010, 2014). However, the influence of the plant’s secondary metabolites on this interaction has not as yet been thoroughly investigated. The abundance of phenylpropanoids in the *A. thaliana* leaf tissue is enhanced when the plant is challenged by *V. longisporum* (König et al., 2014). A time-course study on the effect of the pathogen on tryptophan-derived secondary metabolites revealed an increase in root camalexin concentration and knock-out of biosynthetic genes increased susceptibility to *V. longisporum* (Iven et al., 2012). According to Witzel et al. (2013), certain GLS breakdown products are capable of inhibiting the growth of *V. longisporum*. The four selected *A. thaliana* accessions were chosen on the basis of their diversity in GLS and GLS breakdown product profiles, and were shown to vary with respect to their susceptibility to colonization by the pathogen (**Figure 1**). Although both Bur-0 and Ler-0 emerged as the more tolerant of the four, Häffner et al. (2010, 2014) considered Ler-0 as a sensitive genotype, despite using the same isolate as was employed here. This discrepancy is assumed to have arisen due to the difference in the inoculation method: in Häffner et al.’s (2010, 2014) experiments, plants were uprooted, the roots were trimmed and then exposed to a conidial suspension; whereas the present experiments were based on pouring a conidial suspension on the soil surface, then relying on the natural mode of fungal colonization of the host. Nevertheless, even though Ler-0 was considered here as tolerant on the basis of its biomass accumulation capacity in the face of fungal infection, the abundance of the pathogen within the root of this accession was higher than in the other three accessions, confirming the observation of Häffner et al. (2010) that colonization rate does not correlate with disease severity.

### *Verticillium* Colonization Leads to the Accumulation of GLS in the Roots of Infected Plants

GLS form a part of the plant’s defense against biotic stress (Clay et al., 2009; Aires et al., 2011; Mewis et al., 2012a; Rohr et al., 2012), but few evidential data as yet support a role of GLS in the defense against soil-borne pathogens such as *V. longisporum*. Furthermore, interaction between above and below ground plant tissues in deterring soil-borne pathogens is scarcely investigated. Such systemic examinations should add to a better understanding of the biological basis for GLS variation between shoots and roots, as well as their role in restraining fungal pathogens. Colonization of the host by *V. longisporum* resulted in an increase in the root GLS content in all four accessions, while the GLS response in the leaf was inconsistent. The observed decrease in the case of Bur-0 and Ler-0 may reflect the transport of GLS from the shoot to the root (Madsen et al., 2014) or induced biosynthesis in roots. In Ler-0, the content of 4OHI3M-GLS was significantly downgraded in the leaf, while it accumulated in the root, and the same applied to 7MTH-GLS in Bur-0. It seems possible therefore that the translocation of GLS to the primary infection site contributes to the defense response of these accessions. Further testing of fungal colonization in *A. thaliana* genotypes that are either not able to accumulate GLS in their roots (Andersen et al., 2013) or that are deficient in specific GLS groups (Beekwilder et al., 2008) improve the understanding of the role of GLS in antagonize soil-borne pathogens.

### The Content of GLS Breakdown Products is Reduced by *Verticillium* Colonization

The contribution of GLS to the host defense response has to date focused on the effect of intact GLS compounds (Beekwilder et al., 2008; Markovich et al., 2013), although it is well known that much of the bioactivity of GLS is associated with their breakdown products (reviewed by Hanschen et al., 2014). Analyses of the GLS breakdown product content in the tissues of stressed plants are scarce (Brader et al., 2006; Stotz et al., 2011). The enzymatic degradation of GLS generates a variety of molecules, the bioactivity of most of which still remains obscure. Some breakdown products, in particular the ITCs, have been shown to possess anti-bacterial and/or anti-fungal and/or anti-herbivore properties. The anti-carcinogenic effect of the ITCs is largely due to their ability to covalently bind to nucleophiles such as cysteine residues in tubulin, which inhibits, for example, tubulin polymerization, thus leading to cell growth inhibition and apoptosis induction (Mi et al., 2008). Treatment with a low concentration of ITCs can induce the expression of glutathione *S*-transferases, while at higher concentrations, the hydrogen peroxide generated leads to leaf bleaching (Hara et al., 2010). ITCs have also been implicated in the production of reactive oxygen species and the induction of stomatal closure (Khokon et al., 2011; Hossain et al., 2013), as well as the expression of heat-shock protein genes and an enhanced tolerance to high temperatures stress (Hara et al., 2013); these observations have suggested that ITCs do make a positive contribution to the plant’s abiotic stress tolerance (Martinez-Ballesta et al., 2013). Treatment of *A. thaliana* leaves with 4-(methylsulfinyl)butyl ITC restricts the size of glutathione pool and induces a hypersensitive response (Andersson et al., 2015). Previously, 2Prop-ITC was found growth-inhibiting to *V. longisporum* in a plate assay using freeze-dried *A. thaliana* plant material (Witzel et al., 2013). However, GLS breakdown products differ between lyophilized and fresh plant material due to altered activity of modifying enzymes. In comparison to the earlier findings, formation of 2Prop-ITC was confirmed for Hi-0 but declined for Bur-0. Main degradation product of 2Prop-GLS in leaves was the ITC (Hi-0) or epithionitrile (Bur-0), while CNs were formed in roots (Supplementary Table S2). As *V. longisporum* colonizes the plant via roots, 2Prop-CN levels could be more relevant in deterring fungal spread than 2Prop-ITC, indicating that earlier findings might not account for the *in planta* interaction between host and pathogen. While ITCs predominate among the GLS degradation products, nitriles and epithionitriles are also formed, provided that the necessary enzymes are available. Epithionitriles have been shown to toxic to rat and cattle (Nishie and Daxenbichler, 1980; Collett et al., 2014) and CETB to rats (VanSteenhouse et al., 1999); however, to date the in planta role of nitriles and epithionitriles has been restricted to either herbivore/plant (Lambrix et al., 2001; Mumm et al., 2008) or fungus/plant interactions (Pedras and Hossain, 2011).

Here, the accessions which responded to *V. longisporum* colonization by a reduction in their leaf GLS content (Bur-0 and Ler-0) also experienced a fall in their GLS breakdown product content (and vice versa for Hi-0 and Kn-0, **Figure 4**). The decrease in the leaf nitrile and epithionitrile content noted in Bur-0 and Ler-0 was consistent with the low level of ESP present in these accessions. The behavior of the roots was somewhat unexpected. Except for Hi-0, the content of most of the GLS breakdown products fell in response to the pathogen’s colonization. In the two accessions classed as tolerant (Bur-0 and Ler-0), the production of both nitriles and ITCs was reduced, and in Bur-0, the recovery rate of GLS breakdown products dropped by 50% compared to the rate encountered in the non-infected root. GLS breakdown products have been shown to be antagonistic to the growth of *Verticillium* (Olivier et al., 1999; Down et al., 2004; Njoroge et al., 2011; Witzel et al., 2013), but the biochemical nature of this toxicity remains unclear. In the necrotrophic fungus *Alternaria brassicicola*, exposure to 2Prop-ITC results in the induction of a number of genes associated with the oxidative burst and with cell cycle regulation (Sellam et al., 2007). When *V. longisporum* was presented with xylem sap obtained from oilseed rape, genes encoding both a number of heat-shock proteins and catalase peroxidase were up-regulated; knock-out mutants of these genes resulted in a H_2_O_2_ sensitive phenotype (Singh et al., 2012). Since xylem sap also contains GLS (Andersen et al., 2013) and myrosinase (Floerl et al., 2012), this induction may well be driven by the presence of GLS breakdown products. A working hypothesis for the reduced abundance of GLS breakdown products in infected *A. thaliana* roots is that these compounds interact with fungal targets such as glutathione or tubulin, thereby inhibiting the process of colonization. The product of the *V. longisporum* β-tubulin paralog could represent the relevant target (Inderbitzin et al., 2011). The tolerance of Bur-0, which experienced the most notable decrease in content of GLS breakdown products in the root, may be ascribable to this process.

## Conclusion

Colonization of *A. thaliana* with *V. longisporum* influenced the tissue- and accession-specific accumulation of GLS and their respective breakdown products. Tolerant accessions might be more efficient in accumulating GLS at the infection site in the root. While our study did not identify a particular GLS or breakdown product associated with pathogen tolerance, further examinations should be extended to plants subjected to specific elicitors to accumulate specific GLS prior fungal inoculation (Wiesner et al., 2013a).

## Author Contributions

Conceived and designed the experiments: KW, FH, RK, SR, MS, RG. Performed the experiments: KW, FH, RK. Analyzed the data: KW, FH, RK. Contributed reagents/materials/analysis tools: SR, MS, RG. Wrote the paper: KW, FH, RK, SR, MS, RG.

## Conflict of Interest Statement

The authors declare that the research was conducted in the absence of any commercial or financial relationships that could be construed as a potential conflict of interest.

## Abbreviations

3But-CN: 4-pentenenitrile
3But-GLS: 3-butenyl GLS
3But-ITC: 3-butenyl ITC
CETB: 1-cyano-3,4-epithiobutane
CETP: 1-cyano-2,3-epithiopropane
CETPent: 1-cyano-4,5-epithiopentane
CHETB: 1-cyano-2-hydroxy-3,4-epithiobutane
CN: cyanide
GLS: glucosinolate
I3M-GLS: indole-3-ylmethyl GLS
IAN: 3-indoleacetonitrile
IST: internal standard
ITC: isothiocyanate
4MO-IAN: 4-methoxy-3-indoleacetonitrile
1MOI3M-GLS: 1-methoxyindole-3-ylmethyl GLS
4MOI3M-GLS: 4-methoxyindole-3-ylmethyl GLS
3MSOP-GLS: 3-(methylsulfinyl)propyl GLS
4MSOB-GLS: 4-(methylsulfinyl)butyl GLS
7MSOH-GLS: 7-(methylsulfinyl)heptyl GLS
5MSOP-ITC: 5-(methylsulfinyl)pentylITC
8MSOO-CN: 9-(methylsulfinyl)nonylnitrile
8MSOO-GLS: 8-(methylsulfinyl)octyl GLS
3MTP-CN: 4-(methylthio)butylnitrile
3MTP-GLS: 3-(methylthio)propyl GLS
4MTB-GLS: 4-(methylthio)butyl GLS
4MTB-ITC: 4-(methylthio)butyl ITC
6MTH-CN: 7-(methylthio)heptylnitrile
7MTH-CN: 8-(methylthio)octylnitrile
6MTH-ITC: 6-(methylthio)hexyl ITC
7MTH-ITC: 7-(methylthio)heptyl ITC
7MTH-GLS: 7-(methylthio)heptyl GLS
8MTO-CN: 9-(methylthio)nonylnitrile
8MTO-GLS: 8-(methylthio)octyl GLS
8MTO-ITC: 8-(methylthio)octyl ITC
(*R*)2OH3But-GLS: (*R*)-2-OH-3-butenyl GLS
(*S*)2OH3But-GLS: (*S*)-2-OH-3-butenyl GLS
4OHI3M-GLS: 4-hydroxyindole-3-ylmethyl GLS
3OHP-GLS: 3-hydroxypropyl GLS
4Pent-GLS: 4-pentenyl GLS
4Pent-ITC: 4-pentenyl ITC
2PE-CN: 3-phenylpropanenitrile
2Prop-CN: 3-butenenitrile
2Prop-GLS: 2-propenyl GLS
2Prop-ITC: 2-propenyl ITC
2OH3But-CN: 3-hydroxypentenenitrile
3OHP-GLS: 3-hydroxypropyl GLS
3OHP-CN: 4-hydroxybutylnitrile
3OHP-ITC: 3-hydroxypropyl ITC
n.d.: not detected
To facilitate understanding from which GLS the breakdown products derived: nitriles were abbreviated using the cyanide (CN) name.
