# Supplementary material for: Verticillium longisporum infection induces organ-specific glucosinolate degradation in Arabidopsis thaliana
Source: Front Plant Sci. 2015 Jul 10;6:508. doi: 10.3389/fpls.2015.00508 (PMC4498036; doi:10.3389/fpls.2015.00508)

Supplemental Fig. 1: Variation in the abundance of ESP in the leaves of *A. thaliana*. Plants were either inoculated with *V. longisporum* (+) or non-inoculated (-). Values shown below the immunoblot are based on a densitometric evaluation of the hybridizing bands.


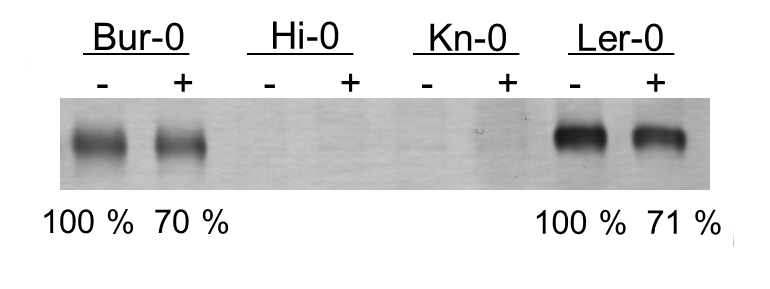

Supplement: Supplementary file 1 [file Data_Sheet_1.DOCX]
