# Supplementary material for: Verticillium longisporum infection induces organ-specific glucosinolate degradation in Arabidopsis thaliana
Source: Front Plant Sci. 2015 Jul 10;6:508. doi: 10.3389/fpls.2015.00508 (PMC4498036; doi:10.3389/fpls.2015.00508)
Supplement: Supplementary file 2 [file Data_Sheet_2.DOCX]

Supplemental Table 1: GLS present in the leaf and root of the four *A. thaliana* accessions Bur-0, Hi-0, Kn-0 and Ler-0, either inoculated or non-inoculated with *V. longisporum*. Mean and SE of GLS content (µmol g^-1^ dry weight) derived from three independent batches of 20 plants, with two technical replicates per sample. Abbreviations are explained in the list of abbreviations.

| Bur-0 |  |  |  |  |
| --- | --- | --- | --- | --- |
| Compound | Leaf Control | Leaf *V. longisporum* | Root Control | Root *V. longisporum* |
| 3OHP-GLS | 0.223 ± 0.145 | 0.409 ± 0.229 | 0.119 ± 0.076 | 0.096 ± 0.081 |
| (*R*)2OH3But-GLS | 0.843 ± 0.059 | 0.726 ± 0.077 | 0.101 ± 0.044 | 0.190 ± 0.062 |
| (*S*)2OH3But-GLS | 2.418 ± 0.059 | 2.056 ± 0.164 | 0.333 ± 0.048 | 0.494 ± 0.200 |
| 2Prop-GLS | 6.707 ± 0.389 | 6.234 ± 0.558 | 0.761 ± 0.066 | 1.430 ± 0.692 |
| 4MSOB-GLS | 0.021 ± 0.011 | 0.010 ± 0.005 | n.d. | 0.002 ± 0.002 |
| 3But-GLS | 10.824 ± 0.495 | 9.881 ± 0.656 | 0.775 ± 0.079 | 1.751 ± 0.939 |
| 4OHI3M-GLS | 0.055 ± 0.010 | 0.040 ± 0.003 | 0.083 ± 0.038 | 0.140 ± 0.067 |
| 3MTP-GLS | 0.103 ± 0.009 | 0.107 ± 0.012 | 0.098 ± 0.026 | 0.124 ± 0.026 |
| 4-Pent-GLS | 0.322 ± 0.010 | 0.298 ± 0.015 | 0.005 ± 0.005 | 0.008 ± 0.008 |
| 7MSOH-GLS | 0.511 ± 0.050 | 0.513 ± 0.054 | 0.282 ± 0.099 | 0.386 ± 0.130 |
| I3M-GLS | 1.601 ± 0.095 | 1.604 ± 0.209 | 0.362 ± 0.044 | 0.562 ± 0.113 |
| 8-MSOO-GLS | 2.319 ± 0.026 | 2.375 ± 0.045 | 1.793 ± 0.410 | 2.481 ± 0.391 |
| 4MOI3M-GLS | 0.804 ± 0.123 | 0.724 ± 0.105 | 0.366 ± 0.123 | 0.534 ± 0.050 |
| 1MOI3M-GLS | 0.057 ± 0.008 | 0.068 ± 0.017 | 1.189 ± 0.130 | 1.678 ± 0.474 |
| 7MTH-GLS | 0.155 ± 0.048 | 0.100 ± 0.044 | 0.163 ± 0.026 | 0.257 ± 0.057 |
| 8MTO-GLS | 0.343 ± 0.100 | 0.308 ± 0.099 | 0.787 ± 0.101 | 1.211 ± 0.242 |
| Hi-0 |  |  |  |  |
| Compound | Leaf Control | Leaf *V. longisporum* | Root Control | Root *V. longisporum* |
| 3MSOP-GLS | 0.118 ± 0.038 | 0.192 ± 0.070 | 0.066 ± 0.006 | 0.185 ± 0.055 |
| 2Prop-GLS | 27.503 ± 3.574 | 29.063 ± 2.536 | 3.772 ± 1.206 | 6.951 ± 1.606 |
| 3But-GLS | 0.108 ± 0.018 | 0.119 ± 0.022 | 0.034 ± 0.020 | 0.057 ± 0.023 |
| 4OHI3M-GLS | 0.024 ± 0.014 | 0.027 ± 0.006 | 0.137 ± 0.100 | 0.132 ± 0.103 |
| 7MSOH-GLS | 0.057 ± 0.029 | 0.093 ± 0.056 | 0.100 ± 0.029 | 0.136 ± 0.025 |
| I3M-GLS | 1.479 ± 0.324 | 1.325 ± 0.240 | 0.500 ± 0.188 | 0.697 ± 0.330 |
| 8-MSOO-GLS | 1.246 ± 0.147 | 1.801 ± 0.364 | 1.384 ± 0.378 | 1.603 ± 0.504 |
| 4MOI3M-GLS | 0.247 ± 0.030 | 0.247 ± 0.068 | 0.314 ± 0.035 | 0.476 ± 0.093 |
| 1MOI3M-GLS | 0.056 ± 0.004 | 0.094 ± 0.035 | 1.283 ± 0.528 | 1.813 ± 0.776 |
| 7MTH-GLS | 0.007 ± 0.007 | 0.009 ± 0.005 | 0.040 ± 0.020 | 0.044 ± 0.025 |
| 8MTO-GLS | 0.175 ± 0.004 | 0.224 ± 0.042 | 0.938 ± 0.331 | 1.200 ± 0.594 |
| Kn-0 |  |  |  |  |
| Compound | Leaf Control | Leaf *V. longisporum* | Root Control | Root *V. longisporum* |
| 3OHP-GLS | 18.157 ± 1.927 | 21.965 ± 3.182 | 5.556 ± 1.580 | 5.296 ± 1.744 |
| 3MSOP-GLS | 0.043 ± 0.025 | 0.091 ± 0.032 | 0.036 ± 0.018 | 0.058 ± 0.043 |
| 4MSOB-GLS | 0.093 ± 0.050 | 0.111 ± 0.049 | 0.074 ± 0.033 | 0.107 ± 0.058 |
| 3But-GLS | 0.023 ± 0.017 | 0.037 ± 0.022 | 0.030 ± 0.022 | 0.041 ± 0.025 |
| 4OHI3M-GLS | 0.018 ± 0.007 | 0.024 ± 0.006 | 0.184 ± 0.092 | 0.260 ± 0.122 |
| 3MTP-GLS | 0.002 ± 0.002 | 0.003 ± 0.003 | 0.006 ± 0.006 | 0.012 ± 0.008 |
| 7MSOH-GLS | 0.081 ± 0.022 | 0.122 ± 0.026 | 0.107 ± 0.045 | 0.128 ± 0.043 |
| 4MTB-GLS | 0.015 ± 0.015 | 0.008 ± 0.008 | 0.053 ± 0.053 | 0.020 ± 0.020 |
| I3M-GLS | 1.917 ± 0.146 | 1.936 ± 0.294 | 0.728 ± 0.156 | 1.225 ± 0.204 |
| 8-MSOO-GLS | 1.137 ± 0.149 | 1.558 ± 0.394 | 2.345 ± 0.326 | 2.777 ± 0.309 |
| 4MOI3M-GLS | 0.581 ± 0.114 | 0.677 ± 0.116 | 0.903 ± 0.178 | 0.745 ± 0.162 |
| 1MOI3M-GLS | 0.078 ± 0.009 | 0.067 ± 0.014 | 1.766 ± 0.376 | 1.919 ± 0.570 |
| 7MTH-GLS | 0.005 ± 0.005 | 0.031 ± 0.019 | 0.123 ± 0.047 | 0.110 ± 0.041 |
| 8MTO-GLS | 0.265 ± 0.068 | 0.376 ± 0.168 | 1.478 ± 0.510 | 2.188 ± 0.840 |
| Ler-0 |  |  |  |  |
| Compound | Leaf Control | Leaf *V. longisporum* | Root Control | Root *V. longisporum* |
| 3OHP-GLS | 18.218 ± 3.663 | 16.930 ± 2.256 | 4.946 ± 1.012 | 2.118 ± 0.908 |
| 3MSOP-GLS | 0.063 ± 0.025 | 0.063 ± 0.040 | 0.077 ± 0.051 | 0.004 ± 0.004 |
| 4MSOB-GLS | 0.060 ± 0.023 | 0.090 ± 0.044 | 0.011 ± 0.011 | 0.006 ± 0.006 |
| 3But-GLS | 0.025 ± 0.025 | 0.028 ± 0.028 | 0.033 ± 0.033 | 0.047 ± 0.047 |
| 4OHI3M-GLS | 0.006 ± 0.003 | 0.009 ± 0.005 | 0.180 ± 0.115 | 0.310 ± 0.176 |
| 3MTP-GLS | 0.116 ± 0.116 | n.d. | 0.005 ± 0.005 | n.d. |
| 7MSOH-GLS | 0.086 ± 0.047 | 0.062 ± 0.021 | 0.104 ± 0.005 | 0.161 ± 0.018 |
| 4MTB-GLS | 0.061 ± 0.061 | 0.014 ± 0.014 | 0.017 ± 0.017 | 0.032 ± 0.032 |
| I3M-GLS | 2.131 ± 0.447 | 2.156 ± 0.393 | 0.364 ± 0.061 | 0.719 ± 0.247 |
| 8-MSOO-GLS | 1.279 ± 0.243 | 1.147 ± 0.144 | 1.542 ± 0.132 | 2.692 ± 0.720 |
| 4MOI3M-GLS | 0.314 ± 0.026 | 0.387 ± 0.065 | 0.358 ± 0.038 | 0.661 ± 0.072 |
| 1MOI3M-GLS | 0.120 ± 0.050 | 0.074 ± 0.011 | 1.234 ± 0.399 | 0.792 ± 0.234 |
| 7MTH-GLS | 0.009 ± 0.009 | 0.007 ± 0.007 | 0.016 ± 0.016 | 0.071 ± 0.036 |
| 8MTO-GLS | 0.172 ± 0.048 | 0.228 ± 0.036 | 0.565 ± 0.171 | 2.085 ± 0.810 |
