# Supplementary material for: Verticillium longisporum infection induces organ-specific glucosinolate degradation in Arabidopsis thaliana
Source: Front Plant Sci. 2015 Jul 10;6:508. doi: 10.3389/fpls.2015.00508 (PMC4498036; doi:10.3389/fpls.2015.00508)
Supplement: Supplementary file 3 [file Data_Sheet_3.DOCX]

Supplemental Table 2: GLS breakdown products in the leaf and root of *A. thaliana* accessions Bur-0, Hi-0, Kn-0 and Ler-0, either inoculated or non-inoculated with *V. longisporum*. Mean and SE of GLS content (µmol g^-1^ fresh weight) derived from three independent batches of 20 plants, with two technical replicates per sample. Abbreviations are explained in the list of abbreviations.

| Bur-0 |  |  |  |  |
| --- | --- | --- | --- | --- |
| Compound | Leaf Control | Leaf *V. longisporum* | Root Control | Root *V. longisporum* |
| 2Prop-CN | 0.0290 ± 0.0051 | 0.0294 ± 0.0059 | 0.0466 ± 0.0258 | 0.0820 ± 0.0368 |
| 2Prop-ITC | 0.0484 ± 0.0128 | 0.0321 ± 0.0154 | 0.0098 ± 0.0050 | 0.0070 ± 0.0041 |
| CEPT | 0.5832 ± 0.0849 | 0.5465 ± 0.0595 | 0.0016 ± 0.0016 | 0.0014 ± 0.0014 |
| 3But-CN | 0.0720 ± 0.0174 | 0.0602 ± 0.0055 | 0.0785 ± 0.0431 | 0.1015 ± 0.0629 |
| 3But-ITC | 0.1273 ± 0.0656 | 0.0787 ± 0.0309 | 0.0952 ± 0.0850 | 0.0076 ± 0.0055 |
| CETB | 1.0310 ± 0.1636 | 0.9823 ± 0.1177 | 0.0004 ± 0.0004 | 0.0025 ± 0.0022 |
| 2OH3But-CN | 0.0219 ± 0.0057 | 0.0253 ± 0.0084 | 0.0103 ± 0.0063 | 0.0112 ± 0.0069 |
| CHETB | 0.1633 ± 0.0308 | 0.1677 ± 0.0315 | n.d. | n.d. |
| 4Pent-ITC | 0.0051 ± 0.0018 | 0.0036 ± 0.0014 | 0.0001 ± 0.0001 | n.d. |
| CETPent | 0.0196 ± 0.0107 | 0.0219 ± 0.0122 | n.d. | n.d. |
| 6MTH-CN | 0.0013 ± 0.0003 | 0.0014 ± 0.0002 | 0.0019 ± 0.0008 | 0.0016 ± 0.0004 |
| 6MTH-ITC | n.d. | n.d. | 0.0072 ± 0.0022 | 0.0055 ± 0.0015 |
| 7MTH-CN | 0.0098 ± 0.0012 | 0.0133 ± 0.0023 | 0.0134 ± 0.0033 | 0.0078 ± 0.0017 |
| 7MTH-ITC | 0.0024 ± 0.0007 | 0.0018 ± 0.0008 | 0.0475 ± 0.0127 | 0.0342 ± 0.0071 |
| 8MTO-CN | 0.0122 ± 0.0014 | 0.0193 ± 0.0028 | 0.0589 ± 0.0135 | 0.0464 ± 0.0145 |
| 8MTO-ITC | 0.0031 ± 0.0012 | 0.0031 ± 0.0013 | 0.2476 ± 0.0624 | 0.2102 ± 0.0553 |
| 8MSOO-CN | 0.0104 ± 0.0036 | 0.0122 ± 0.0025 | 0.0096 ± 0.0060 | n.d. |
| 8MSOO-ITC | 0.0007 ± 0.0007 | n.d. | 0.0007 ± 0.0006 | n.d. |
| 2PE-CN | 0.0077 ± 0.0014 | 0.0082 ± 0.0011 | n.d. | n.d. |
| 2PE-ITC | 0.0010 ± 0.0005 | 0.0007 ± 0.0004 | 0.0001 ± 0.0001 | n.d. |
| IAN | 0.0234 ± 0.0090 | 0.0203 ± 0.0052 | 0.0190 ± 0.0042 | 0.0198 ± 0.0050 |
| 4MO-IAN | 0.0045 ± 0.0027 | 0.0044 ± 0.0012 | 0.0034 ± 0.0013 | 0.0037 ± 0.0011 |

| Hi-0 | |  | |  | |  | |  | |
| --- | --- | --- | --- | --- | --- | --- | --- | --- | --- |
| Compound | | Leaf Control | | Leaf *V. longisporum* | | Root Control | | Root *V. longisporum* | |
| 2Prop-CN | | 0.3956 ± 0.1047 | | 0.4480 ± 0.0677 | | 0.2248 ± 0.1697 | | 0.4352 ± 0.2809 | |
| 2Prop-ITC | | 0.8843 ± 0.0542 | | 0.9674 ± 0.0507 | | 0.0203 ± 0.0071 | | 0.0234 ± 0.0108 | |
| 3But-CN | | n.d. | | n.d. | | 0.0092 ± 0.0092 | | n.d. | |
| 2OH3But-CN | | n.d. | | n.d. | | 0.0004 ± 0.0004 | | 0.0004 ± 0.0004 | |
| 6MTH-CN | | n.d. | | n.d. | | 0.0003 ± 0.0003 | | n.d. | |
| 6MTH-ITC | | n.d. | | n.d. | | 0.0013 ± 0.0006 | | 0.0002 ± 0.0002 | |
| 7MTH-CN | | n.d. | | n.d. | | 0.0023 ± 0.0014 | | 0.0016 ± 0.0006 | |
| 7MTH-ITC | | n.d. | | n.d. | | 0.0155 ± 0.0041 | | 0.0130 ± 0.0031 | |
| 8MTO-CN | | n.d. | | n.d. | | 0.0294 ± 0.0098 | | 0.0399 ± 0.0154 | |
| 8MTO-ITC | | 0.0010 ± 0.0007 | | 0.0016 ± 0.0008 | | 0.1985 ± 0.0596 | | 0.2785 ± 0.0868 | |
| 8MSOO-CN | | n.d. | | n.d. | | 0.0010 ± 0.0010 | | 0.0006 ± 0.0006 | |
| 8MSOO-ITC | | 0.0002 ± 0.0002 | | 0.0002 ± 0.0002 | | 0.0006 ± 0.0006 | | n.d. | |
| IAN | | 0.0004 ± 0.0004 | | 0.0009 ± 0.0006 | | 0.0238 ± 0.0086 | | 0.0137 ± 0.0027 | |
| 4MO-IAN | | n.d. | | n.d. | | 0.0020 ± 0.0007 | | 0.0027 ± 0.0008 | |
| Kn-0 | |  | |  | |  | |  | |
| Compound | | Leaf Control | | Leaf *V. longisporum* | | Root Control | | Root *V. longisporum* | |
| 3OHP-CN | | 0.0532 ± 0.0231 | | 0.0923 ± 0.0468 | | 0.0621 ± 0.0393 | | 0.0064 ± 0.0064 | |
| 3OHP-ITC | | 1.3435 ± 0.4474 | | 1.5309 ± 0.3808 | | 0.1031 ± 0.0609 | | 0.0193 ± 0.0118 | |
| 6MTH-ITC | | n.d. | | n.d. | | 0.0002 ± 0.0002 | | n.d. | |
| 7MTH-CN | | n.d. | | n.d. | | 0.0068 ± 0.0034 | | 0.0043 ± 0.0025 | |
| 7MTH-ITC | | n.d. | | n.d. | | 0.0049 ± 0.0030 | | 0.0095 ± 0.0049 | |
| 8MTO-CN | | n.d. | | n.d. | | 0.1001 ± 0.0486 | | 0.0779 ± 0.0438 | |
| 8MTO-ITC | | 0.0054 ± 0.0008 | | 0.0063 ± 0.0008 | | 0.1248 ± 0.0702 | | 0.2225 ± 0.1204 | |
| 8MSOO-CN | | 0.0019 ± 0.0019 | | 0.0016 ± 0.0016 | | 0.0196 ± 0.0191 | | 0.0002 ± 0.0002 | |
| 8MSOO-ITC | | 0.0022 ± 0.0022 | | 0.0020 ± 0.0020 | | 0.0025 ± 0.0025 | | n.d. | |
| IAN | | 0.0007 ± 0.0005 | | 0.0018 ± 0.0015 | | 0.0239 ± 0.0096 | | 0.0149 ± 0.0054 | |
| 4MO-IAN | | 0.0006 ± 0.0006 | | 0.0004 ± 0.0004 | | 0.0037 ± 0.0019 | | 0.0013 ± 0.0004 | |
| Ler-0 | |  | |  | |  | |  | |
| Compound | | Leaf Control | | Leaf *V. longisporum* | | Root Control | | Root *V. longisporum* | |
| 3OHP-CN | | 0.4969 ± 0.1043 | | 0.3232 ± 0.0433 | | 0.0430 ± 0.0286 | | 0.0189 ± 0.0189 | |
| 3OHP-ITC | | 0.1579 ± 0.0953 | | 0.1128 ± 0.0333 | | 0.0820 ± 0.0317 | | 0.0322 ± 0.0270 | |
| 7MTH-CN | | 0.0007 ± 0.0004 | | 0.0001 ± 0.0001 | | 0.0004 ± 0.0004 | | 0.0002 ± 0.0002 | |
| 7MTH-ITC | | 0.0001 ± 0.0001 | | n.d. | | 0.0086 ± 0.0024 | | 0.0091 ± 0.0022 | |
| 8MTO-CN | | 0.0088 ± 0.0004 | | 0.0030 ± 0.0002 | | 0.0430 ± 0.0102 | | 0.0234 ± 0.0048 | |
| 8MTO-ITC | | 0.0019 ± 0.0012 | | 0.0016 ± 0.0009 | | 0.2294 ± 0.0605 | | 0.1822 ± 0.0422 | |
| 8MSOO-CN | | 0.0067 ± 0.0067 | | 0.0021 ± 0.0021 | | 0.0057 ± 0.0053 | | 0.0174 ± 0.0171 | |
| 8MSOO-ITC | | 0.0005 ± 0.0005 | | n.d. | | 0.0006 ± 0.0006 | | 0.0017 ± 0.0017 | |
| IAN | | 0.0323 ± 0.0096 | | 0.0218 ± 0.0050 | | 0.0133 ± 0.0056 | | 0.0132 ± 0.0068 | |
| 4MO-IAN | | 0.0028 ± 0.0002 | | 0.0017 ± 0.0001 | | 0.0020 ± 0.0015 | | 0.0020 ± 0.0016 | |
